# The multifaceted therapeutic value of targeting steroid receptor coactivator-1 in tumorigenesis

**DOI:** 10.1186/s13578-024-01222-8

**Published:** 2024-03-29

**Authors:** Qiang Chen, Peng Guo, Yilin Hong, Pingli Mo, Chundong Yu

**Affiliations:** 1 Zhejiang Key Laboratory of Pathophysiology, Department of Biochemistry and Molecular Biology, Health Science Center, Ningbo University, Ningbo, Zhejiang 315211 China; 2 Key Laboratory of Precision Medicine for Atherosclerotic Diseases of Zhejiang Province, Affiliated First Hospital of Ningbo University, Ningbo, Zhejiang 315010 China; 3 Department of Cell Biotechnology Laboratory, Tianjin Cancer Hospital Airport Hospital, Tianjin, 300308 China; 4 State Key Laboratory of Cellular Stress Biology, Innovation Center for Cell Biology, School of Life Sciences, Xiamen University, Xiamen, Fujian 361104 China

**Keywords:** SRC-1, NCOA1, Steroid hormone, Tumour progression

## Abstract

Steroid receptor coactivator-1 (SRC-1, also known as NCOA1) frequently functions as a transcriptional coactivator by directly binding to transcription factors and recruiting to the target gene promoters to promote gene transcription by increasing chromatin accessibility and promoting the formation of transcriptional complexes. In recent decades, various biological and pathological functions of SRC-1 have been reported, especially in the context of tumorigenesis. SRC-1 is a facilitator of the progression of multiple cancers, including breast cancer, prostate cancer, gastrointestinal cancer, neurological cancer, and female genital system cancer. The emerging multiorgan oncogenic role of SRC-1 is still being studied and may not be limited to only steroid hormone-producing tissues. Growing evidence suggests that SRC-1 promotes target gene expression by directly binding to transcription factors, which may constitute a novel coactivation pattern independent of AR or ER. In addition, the antitumour effect of pharmacological inhibition of SRC-1 with agents including various small molecules or naturally active compounds has been reported, but their practical application in clinical cancer therapy is very limited. For this review, we gathered typical evidence on the oncogenic role of SRC-1, highlighted its major collaborators and regulatory genes, and mapped the potential mechanisms by which SRC-1 promotes primary tumour progression.

## Introduction

Cellular signal transduction and physiological function regulation in living organisms require special messenger networks in which steroid materials, including sex hormones, thyroid hormones, glucocorticoids, etc., are critical intermediates [1]. Steroids serve as messengers in gene transcriptional regulation, which requires the cooperation of their nuclear receptors (NRs) and coactivators; NRs, such as estrogen receptor (ERα/β), glucocorticoid (GR), androgen receptor (AR), and thyroid receptor (TR), are ligand-inducible transcription factors, and their transcriptional activity requires the collaboration of corresponding coactivators [1, 2]. Upon activation, NRs undergo dimerization, subsequently recruiting corresponding coactivators via their activation function 2 (AF2) domains, and the AF2 domain links the LXXLL motifs in the nuclear receptor interaction domain (NRID) of coactivators [3].

**Fig. 1:**
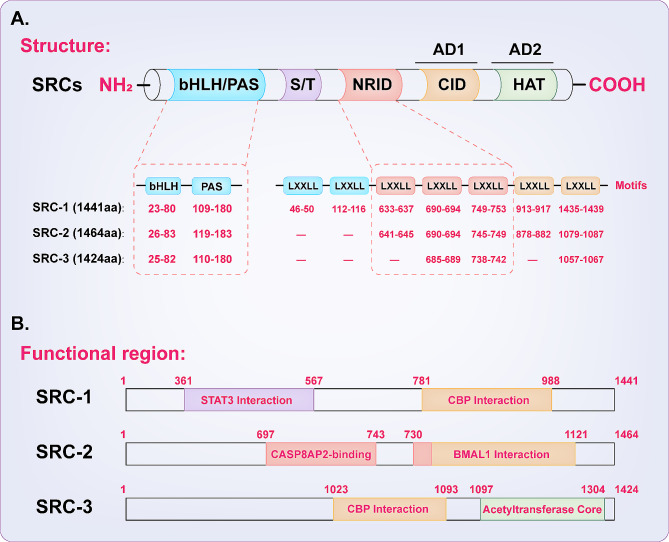
Structures and functional regions of steroid receptor coactivators. (**A**) The domain structure diagram of steroid receptor coactivators. (**B**) The functional regions of steroid receptor coactivators. Three homologous SRC proteins (i.e., SRC-1, SRC-2, and SRC-3) have similar molecular weights and protein domains, including bHLH-PAS, NRID AD1, and AD2 domains. The LXXLL motifs located on the NRID are responsible for direct interaction with NRs, and the ADs are primarily responsible for promoting the formation of transcriptional complexes by recruiting secondary coregulatory factors, including p300, CBP, PRMT1, and CARM1

Steroid receptor coactivators (SRCs; also known as nuclear receptor coactivators, NCOAs), a member of the p160 family, are crucial for increasing the transcriptional activity of the steroid-NR axis. The SRC gene family contains three homologues, namely, SRC-1 (NCOA1), SRC-2 (NCOA2/TIF2), and SRC-3 (NCOA3/AIB1), which encode proteins that are similar in molecular weight (approximately 160 kDa). Their amino acid sequences and protein domains are also similar. Structurally, the SRC members share three functional domains with high degrees of similarity, including an N-terminal basic helix-loop-helix/Per/ARNT/Sim domain (bHLH-PAS), an NRID and two C-terminal activation domains (AD1 and AD2) (Fig. 1A) [4, 5]. The bHLH-PAS domains of the three SRC members are slightly different but highly conserved and contain a bipartite nuclear localization sequence (NLS). The NRID contains 2 or 3 LXXLL motifs (X represents any amino acid) that are responsible for direct interactions with NRs, and the overall distribution of these particular motifs is distinct. The AD is primarily responsible for promoting the formation of transcriptional complexes by recruiting secondary coregulatory factors, including histone acetyltransferase p300, cointegrators such as CREB-binding protein (CBP), protein arginine N-methyltransferase 1 (PRMT1), and coactivator-associated arginine methyltransferase 1 (CARM1) (Fig. 1B) [5]. The CBP/p300 complex recruited by AD1 is crucial for SRC-mediated transcriptional activation, and the corresponding SRC domain is called the CBP interacting domain (CID). PRMT1 and CARM1 histone methyltransferases can be recruited by AD2, which is located at the C-terminus of SRCs. Previous studies revealed that an intrinsic histone acetyltransferase (HAT) core is also present in this region [6, 7], but the evidence related to the HAT activity of SRCs is still insufficient.

The coactivator role of SRC members is essential for the full transcriptional activation of the steroid receptor superfamily. In 1995, Oñate and colleagues first discovered that SRC-1 could interact with progesterone receptor (PR) and promote its transcriptional activation [8]; subsequently, SRC-2 [9] and SRC-3 [7, 10–13] were identified. In the past 30 years, more than 300 transcriptional coactivators have been reported [14], and as an early identified coactivator, SRC-1 has been widely shown to play roles in various biological and pathological processes, including lipid metabolism and transport, neuronal synaptic plasticity, energy homeostasis, vascular endothelial injury, inflammation, diabetes progression, and tumorigenesis. Nonetheless, new information on the oncogenic role of SRC-1 has emerged in recent years. Here, we mainly review the notable role of SRC-1 in promoting tumour progression, particularly, its coactivation partners in several tissues and pathological states.

## SRC-1 serves as a transcriptional coactivator to promote gene transcription

**Fig. 2:**
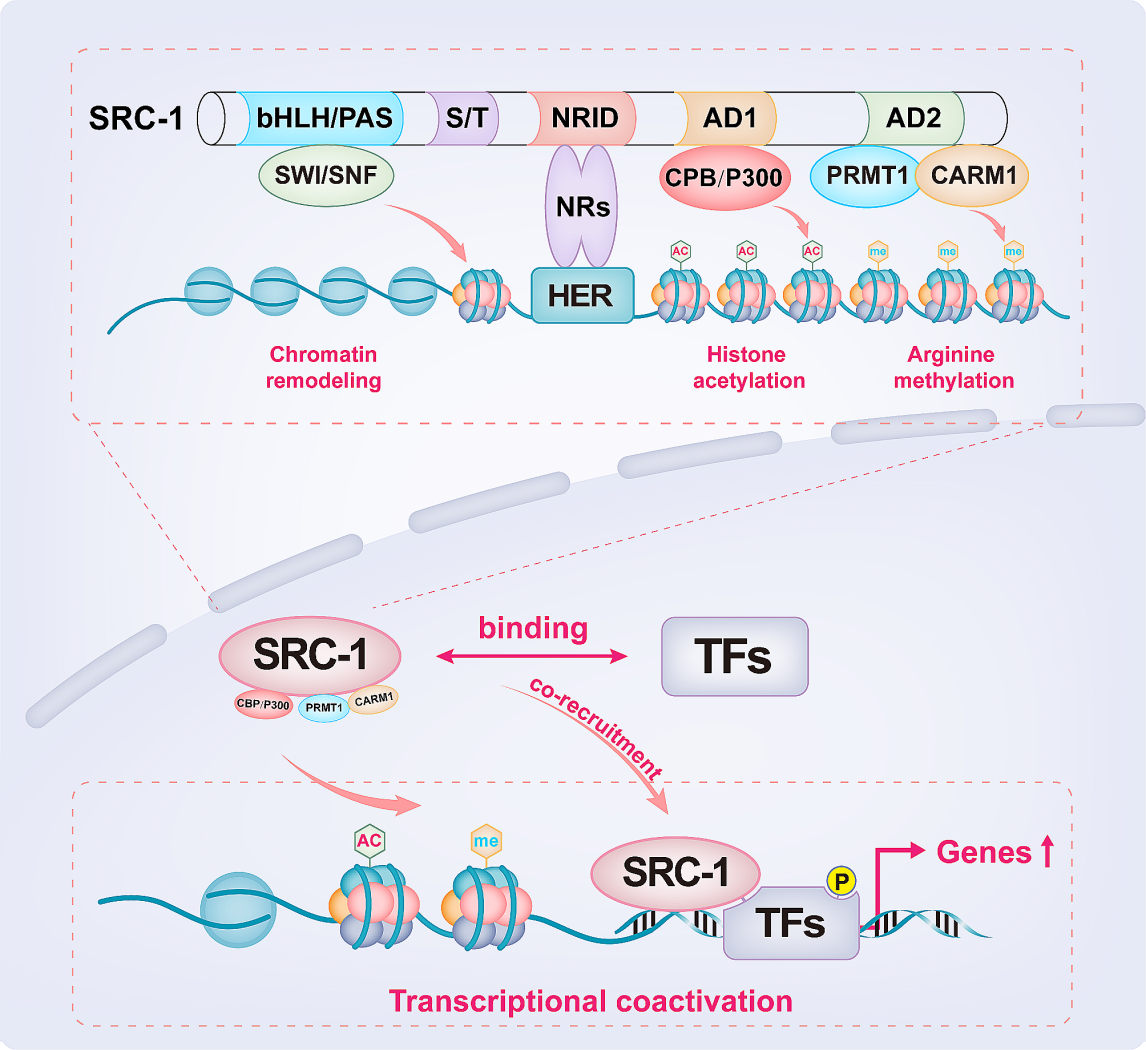
The pattern of SRC-1 coactivation. The classical pattern is that SRC-1 serves as a coactivator in a nuclear receptor-dependent, non-DNA binding manner; SRC-1 induces structural changes in nuclear receptors and recruits CBP/P300, PRMT1, and CARM1 to form a transcriptional complex. The emerging model is that SRC-1 directly binds to TFs and co-recruits to the target gene promoter to promote target gene transcription

The prevailing view is that steroid hormones require corresponding receptors and coactivators to transmit activation signals to regulate physiological functions, and SRC-1 is a crucial regulator. The classical view is that SRC-1 serves as a coactivator in a nuclear receptor-dependent non-DNA binding pattern in which SRC-1 induces structural changes in steroid receptors (or nuclear receptors) that are critical for transcriptional activation (Fig. 2, upper part). The bHLH/PAS domain located at the N-terminus contains signalling peptides that guide the nuclear transport of SRC-1, and this region also contains a binding site for the SWI/SNF complex, which is crucial for an open chromatin structure [15]. The NRID is responsible for identifying and binding NR and transmitting transcriptional activation signals, while AD1 or AD2 located at the C-terminus can recruit histone acetyltransferases (e.g., CBP/P300) or methylases (e.g., PRMT1 and CARM1) to form a transcriptional complex. As previously mentioned, histone acetylation by CBP/P300 or histone arginine methylation by PRMT1 and CARM1 both increase transcriptional activity [5], which may be the core mechanism of SRC-1 coactivation. Moreover, an emerging pattern in which SRC-1 synergistically promotes target gene transcription by directly binding to transcription factors has been proposed and supported by increasing evidence (Fig. 2, lower part) [16].

## SRC-1 has diverse biological functions

**Fig. 3:**
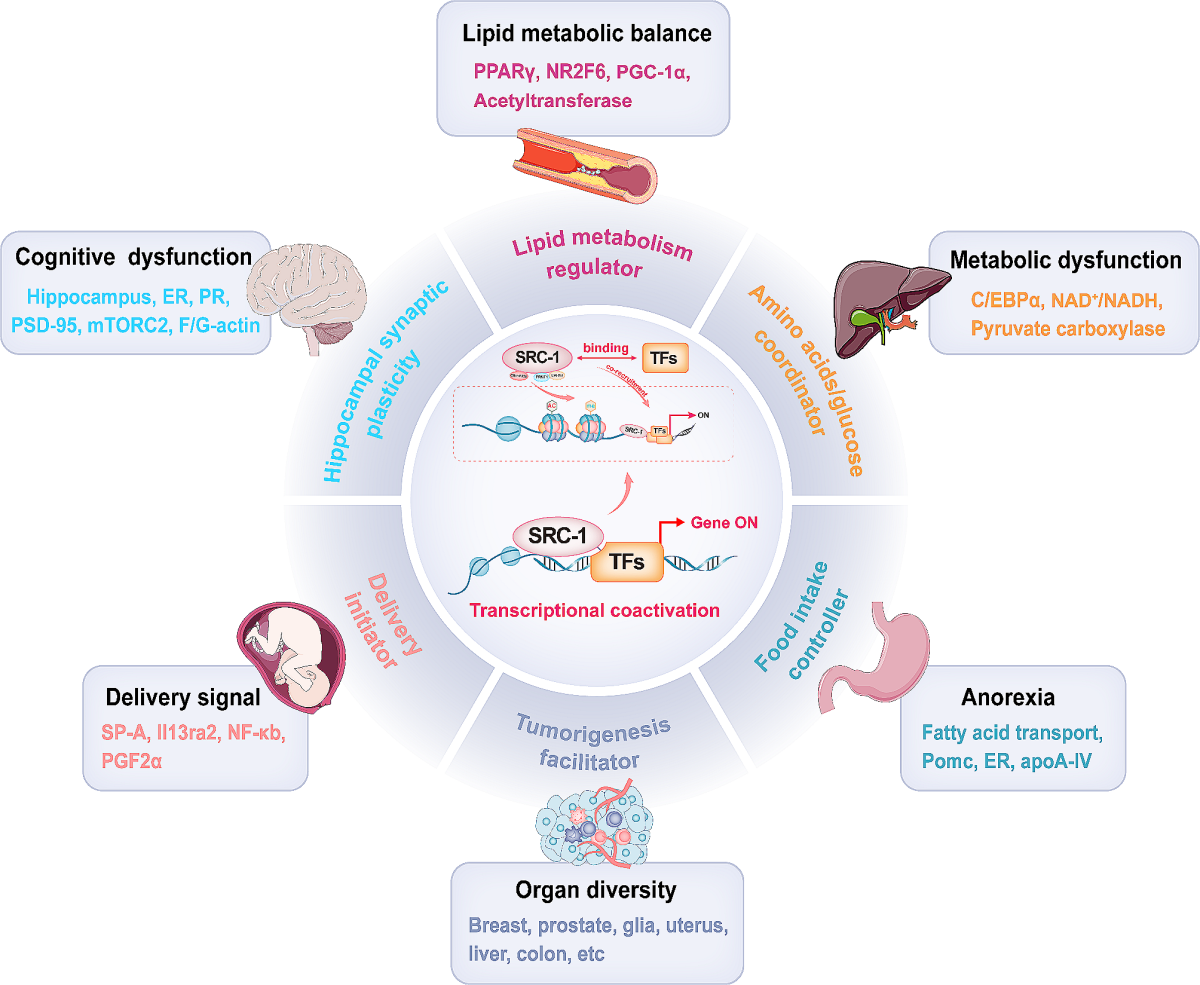
The role of SRC-1 in promoting various biological and pathological processes. SRC-1 has various biological functions and serves as a coactivator for various genes that regulatemetabolic homeostasis, food intake, learning/memory, parturition, and tumorigenesis. SRC-1 is recognized as a coactivator of PPARγ, NR2F6, PGC-1α, and acetyltransferase and participates in the regulation of genes involved in lipid metabolism and adipocyte differentiation. SRC-1 transactivates pyruvate carboxylase by elevating the expression of C/EBPα and regulates both glucose and NAD^+^/NADH homeostasis, thus participating in the Warburg effect. SRC-1 promotes leptin-mediated STAT3 depolarization and Pomc expression, participating in regulating food intake. In the brain, SRC-1 regulates ER-mediated induction of PR-related gene expression and plays a key role in regulating hippocampal synaptic plasticity and spatial learning and memory. Additionally, SRC-1 promotes parturition by regulating the expression of several genes, including *SP-A*, partner genes of NF-κb activation, *PGF2α*, and *Il13ra2*

The role of SRC-1 in promoting various biological and pathological processes, including metabolic homeostasis, food intake, learning/memory, and parturition, has been reported, so its nontumorigenic roles were reviewed (Fig. 3).

### SRC-1 modulates metabolic homeostasis

Peroxisome proliferator-activated receptors (PPARs) are key participants in lipid metabolic homeostasis. SRC-1 is recognized as a coactivator of PPARγ that enhances ligand-dependent transcription to participate in the regulation of genes involved in lipid metabolism and adipocyte differentiation [17, 18], and the ligand binding domain (LBD) of PPARγ is critical for SRC-1 recruitment [19, 20]. However, the relationship between SRC-1 and PPARα remains controversial. Previous studies have indicated that SRC-1 is not essential for PPARα-regulated gene expression [21], but evidence from a dynamic fluorescence tracking system suggests that SRC-1 can bind to PPARα [22]. The metabolic balance of lipids is dynamically regulated by the SRC-2/SRC-1 ratio, which is highly sensitive to high-fat diets [23]. Genetic deletion of SRC-2 reduced the activity of PPARγ, suppressed white adipose accumulation and accelerated the thermogenesis of PGC-1 in brown adipose tissue by promoting the interaction between SRC-1 and PGC-1α, whereas genetic deletion of SRC-1 triggered energy metabolic dysfunction and obesity [23]. Another study indicated that knockdown of SRC-1 in the nucleus solitary tract (NTS) of rats suppressed the 17β-estradiol-induced anorectic action, which in turn increased the animals’ food intake and body weight [24]. Apolipoprotein A-IV (apoA-IV), which is crucial for fatty acid transport, can be upregulated by 17β-estradiol-nuclear receptor signalling, in which SRC-1 serves as an indispensable coactivator that increases transcriptional activity [24]. They also found that high-fat feeding could downregulate the expression of SRC-1 in ovariectomized rats [24], whereas Yamamuro et al. reported that the expression of multiple adipogenic genes, including SRC-1 and SRC-2, was attenuated in the adipose tissue of fasted mice [25]. SRC-1 promotes hepatic steatosis via NR2F6. SRC-1 can be recruited to the promoter of the fatty acid translocase CD36 when NR2F6 binds this promoter, so these factors jointly promote its histone acetylation to increase its transcriptional activity [26]. Furthermore, SRC-1 is considered to be an acetyltransferase and is involved in obesity-related vascular disease progression. The adaptor p66^Shc^, which is part of a complex mitochondrial system, regulates endogenous ROS generation to drive vascular injury, and the histone methyltransferase SUV39H1 orchestrates the recruitment of JMJD2C and SRC-1 to the promoter of the p66^Shc^ adaptor, resulting in decreased H3K9me2/3 levels and elevated H3K9ac levels, which promote gene transcription [27]. However, SRC-1 may not have acetyltransferase activity, and its binding to the CBP/p300 complex is key for this activity [5].

SRC-1 is involved in amino acid metabolic homeostasis and gluconeogenesis, and the dysregulation of these processes is a risk factor for chronic diseases such as diabetes and cancer. SRC-1 can modulate tyrosine biosynthesis in the liver by regulating the transcriptional activity of tyrosine aminotransferase (TAT) [28]. Moreover, SRC-1-deficient mice exhibit overall impairment of amino acid metabolism due to low TAT levels, as well as hyperthyroxinemia and corneal alterations, which are two clinical features of human TAT deficiency syndrome [28]. SRC-1 is an essential coordinator of hepatic glucose production, and SRC-1-deficient mice develop hypoglycaemia due to insufficient hepatic glucose production; moreover, conditional expression of SRC-1 in the liver rescues the blood glucose levels in mice [29]. Pyruvate carboxylase is a catalytic enzyme cruvial for the initiation of gluconeogenesis, and SRC-1 can transactivate pyruvate carboxylase by increasing the expression of C/EBPα [29]. In addition, SRC-1 serves as a regulator of both glucose and NAD^+^/NADH homeostasis, thus participating in the Warburg effect in tumour cells [30]. In the absence of glucose, SRC-1 can be stabilized by the 26 S proteasome and promotes the conversion of NADH to NAH^+^ by upregulating the expression of complex I of the mitochondrial electron transport chain [30].

### SRC-1 plays a key role in synaptic plasticity and energy homeostasis in neurons

Recent reports suggest that SRC-1 can also indirectly modulate energy metabolism by affecting food intake. Leptin is a signal of nutritional deficiency, and decreasing leptin levels trigger a range of responses, including restoring energy homeostasis by increasing food intake and reducing energy expenditure [31]. During eating, an increase in leptin levels triggers neuronal activation and expression of the anorexic peptide Pomc, leading to a decrease in food intake. Genetic deletion of SRC-1 in mice attenuated leptin-mediated STAT3 depolarization and Pomc expression, which promoted food intake and high-fat diet-induced obesity in mice [31, 32].

In addition, SRC-1 plays a key role in regulating hippocampal synaptic plasticity and spatial learning and memory [33, 34]. In the brain, SRC-1 functions to regulate ER-mediated PR gene induction and hormone-dependent behaviour, and there are sex-specific differences in its expression in specific regions of the brain [35–38]. The age-related differences in SRC-1 expression are also noteworthy, as the level of SRC-1 significantly decreases with age in various brain functional regions, including those related to motor centre regulation, learning and memory, and in neural stem cells [39]. Aromatase plays an important role in the regulation of hippocampus-related learning, memory, and cognitive functions by catalysing the conversion of androgen to estrogen in the hippocampus. It has been reported that the aromatase inhibitor letrozole can inhibit the expression of SRC-1 in functional areas of the brain, which may induce neurological disorders [40]; in addition, SRC-1 may modulate hippocampal synaptic plasticity by regulating synaptic protein PSD-95 expression and estrogen signalling [41]. Estrogen regulates actin polymerization and spatial memory in the hippocampus by activating estrogen receptor α/β and SRC-1-mammalian target of rapamycin complex 2 (mTORC2) signalling [42–44]. Another study showed that letrozole regulated the F/G-actin ratio in the hippocampus of mice by inhibiting the expression of SRC-1, and this effect was reversed by estrogens [45]. These results suggest that SRC-1 plays an important role in regulating actin cytoskeleton dynamics and local estrogens-mediated synaptic plasticity in the hippocampus. Moreover, SRC-1 expression was significantly downregulated in the hippocampus of mice who underwent orchiectomy (ORX), and this effect could be reversed by testosterone in a dose-dependent manner [46]. However, the effect of testosterone on cognitive maintenance seems to be insignificant in comparison to the effect of local estrogens [47]. Undoubtedly, SRC-1 is closely related to cognition and is a major risk factor for Alzheimer’s disease (AD).

### SRC-1 contributes to the onset of mammalian parturition

Surfactant protein-A (SP-A), which is secreted into amniotic fluid by the foetal lung, is considered the initiation signal of parturition, and SRC-1/2 can promote the initiation of parturition by upregulating SP-A expression [48, 49]. Parturition was severely delayed in heterozygous maternal mice harbouring SRC-1/-2-deficient embryos, and the expression of key genes involved in parturition, including the partner genes of NF-κb activation, *PGF2α*, and contraction-related genes, which are associated with impaired luteolysis and high circulating progesterone, was reduced in the myometrium of the maternal uterus [49]. Clinically, the expression of SRC-1 in the foetal membranes of patients predelivery was greater than that of patients postdelivery [50]. Progesterone participates in the regulation of early pregnancy by inhibiting estrogen-induced cell proliferation and inducing stromal cell differentiation during decidualization to promote endometrial receptivity; interleukin-13 receptor subunit alpha-2 (Il13ra2) is the executor of this process and is regulated by SRC-1 [51].

SRC-1 can also participate in the dynamic regulation of blood pressure. The evidence from the Framingham Heart Study showed that a single-nucleotide polymorphism of the SRC-1 gene (rs1550383) is associated with elevated diastolic blood pressure in women but not in men [52]. Compared with wild-type littermates, female mice with genetic deletion of SRC-1 exhibited increased blood pressure and aortic stiffness, and SRC-1-deficient mice exhibited increased cardiac energy expenditure [53]. Hyperglycaemia-induced endothelial cell injury is the major trigger for the development of cardiovascular disease, and SRCs are related to the regulation of vascular homeostasis; SRC-1 is expressed in endothelial cells, vascular smooth muscle cells, and neointimal cells and promotes vascular protection by inhibiting the formation of neointima after vascular injury [54, 55].

## SRC-1 is a crucial promoter of the progression of various tumours

The carcinogenic role of SRC-1 was discovered after its identification in 1995. Initially, SRC-1 was found to serve as a predictor of tamoxifen response in recurrent breast cancer patients [56]; it is an independent predictor of reduced disease-free survival (DFS) [57]. Its novel role in promoting the progression and metastasis of various tumours, particularly tumours of the reproductive and urinary systems, including tumours of the breast, prostate, uterus, and ovary, has since been reported (Table 1). These organs, which contain abundant steroid hormones, are the epicentre of SRC-1 dysregulation, as secreted sex hormones or glucocorticoids regulate transcription and physiological function through their nuclear receptors and rely on the assistance of SRC-1.

**Table 1:** The novel role of SRC-1 in promoting various tumor progression

Tissue	Collaborator	Regulated genes	Transcriptional potential	Ref
Breast	SRC-3	—	Interacting with ER to regulate the core genes of breast cancer progression	[58, 59]
	Cyclin D1	—	Cyclin D1 serves as a bridging factor between ER and SRC-1	[60]
	Ets2	c-Myc, MMP9, HER2, CSF-1	Interacting with Ets2 to upregulate target gene expression	[61, 68–70]
	HOXC11	S100beta	Collaborate with HOXC11 to promote S100beta expression	[63]
	—	ADAM22	Promoting steroid resistance by upregulating ADAM22 expression	[62]
	Methylation factor complex	NR2F2, NTRK2, SETBP1, CTDP1, POU3F2	Promoting endocrine resistance through an epigenetics reprogramming pathway	[64]
	STAT1	SMAD2, ASCL1, NFIA, E2F7	Binding with STAT1 to promote its transcriptional activity	[65]
	Ets1/2	HER2	Ets1/2 upregulates HER2 expression by recruiting SRC-1	[67].
	c-Fos	CSF-1	SRC-1 and c-Fos can be recruited to the functional AP-1 site in the CSF-1 promoter	[71]
	c-Fos, HIF1α	VEGFa	Combining and recruiting the AP-1 site or HIF1α-binding element of VEGFa promoter	[72]
	PEA3	Twist, N-cadherin, vimentin	Collaborating with PEA3 to promote Twist expression to regulate target genes	[73]
	AP-1	ITGA5	Collaborating with AP-1 to promote transcriptional activity of ITGA5	[74]
	STAT3	Leptin mediated genes	Interacting with the STAT3 activation domain to enhance its signal transduction	[75].
Prostate Cancer	AR, Androgen, IL-6, MAPK	AR-regulated genes	Enhancing the transactivation of AR	[76–78]
	AR	PSA	Promoting the proliferation of prostate cancer cells by regulating PSA	[79, 80]
	RORγ	AR-regulated genes	Be recruited to AR-ROR response elements by RORγ	[82]
Endometrial cancer	—	Mig-6	SRC-1 is reduced in endometrial cancer tissue	[97, 98]
Ovarian cancer	ERα, Estrogens	c-Myc	c-Myc is downregulated in SRC-1-deficient cells	[99]
Meningioma	Progesterone, PR	PR-regulated genes	SRC-1 is expressed in the tissues of meningioma patients	[88]
Astrocytoma	Progesterone, PR	VEGF	VEGF downregulated in SRC-1 deficient cells	[89, 91].
	Estradiol, ERα	—	Promoting estradiol induced astrocytoma cell growth	[92]
Glioblastoma	bFGF, PEA3	VEGF, MMP9	Enhancing bFGF or PEA3 mediated angiogenesis	[93]
	XIST/mi-152	KLF4	Promoting KLF4 expression and cell glioma of glioblastoma	[94]
Liver cancer	β-catenin	c-Myc, PCNA	Interacting with β-catenin directly to enhance Wnt/β-catenin signaling	[83, 84]
	miR-105-1	—	miR-105-1 negatively regulates the mRNA level of SRC-1	[85]
Colorectal cancer	miR-4443	—	miR-4443 negatively regulates the mRNA level of SRC-1	[86]
	GLI2	cyclin D1, Bcl-2, Slug, N-cadherin, Vimentin	Enhancing Hedgehog signaling by directly binding to the zinc finger domain of GLI2	[16]
Melanoma	HOXC11	S100beta	Collaborating with HOXC11 to promote S100beta expression	[100]
Thyroid cancer	NF-κB	VEGFC	Combining with NF-κB to form a coactivating complex that directly promotes VEGFC transcription	[101, 102]

### Breast cancer

Acquired resistance to endocrine therapy in breast cancer is a major clinical challenge, where the involvement of SRC-1 in estrogen receptor (ER)-mediated resistance is critical. ER is an important regulator of mammary epithelial growth and differentiation, and its transactivation is dependent on leucine-rich motifs, which constitute the ligand-regulated binding site of SRC-1. ER contains α and β subunits that can bind to estrogen and act as homodimers or heterodimers to bind to the estrogen responsive element (ERE) of the target gene; SRC-1 and SRC-3 can be heterodimerized and recruited to the promoter of genes containing classical EREs [58].

The ER can interact with SRC-1 to modulate the expression of genes central to breast cancer progression, but the expression of its beta subunit is negatively correlated with that of SRC-1 [59]. Cyclin D1 can act as a bridging factor between the ER and SRC-1, recruiting SRC-1 to the ER in the absence of ligands to promote breast cancer progression [60]. However, ER-dependent transcription is not limited to the regulation of SRC-1; for example, c-Myc expression is blocked in SRC-1-deficient MCF7 cells but can be rescued with estrogen stimulation [61]. Some studies also indicate that the resistance of breast cancer to endocrine therapy is due to an increase in cell plasticity, which leads to the emergence of hormone-independent tumours. SRC-1 can drive tumour adaptation by interacting with developmental proteins and other nonsteroidal transcription factors [62]. HOXC11 is a regulator of cellular development, and SRC-1 cooperates with HOXC11 to promote the expression of the calcium-binding protein S100beta in endocrine-resistant breast cancer cells [63]. ADAM22, which is highly expressed in endocrine-resistant tumours, plays a critical role in the SRC-1-mediated transition of steroid-responsive tumours to a steroid-resistant state [62]. Another study demonstrated that SRC-1 modulates endocrine-resistant breast cancer progression through an epigenetic reprogramming pathway, and a set of prodifferentiation genes associated with poor clinical outcome (i.e., *NR2F2*, *NTRK2*, *SETBP1*, *CTDP1*, and *POU3F2*) were found to be hypermethylated by SRC-1 combined with a complex of methylators [64]. In addition, SRC-1 has been reported to mediate endocrine resistance independent of ER receptors; in this context, STAT1 acts a novel transcription factor partner of SRC-1. SRC-1 directly binds to STAT1 to initiate a transcriptional cascade and promote the expression of a set of central endocrine resistance-related genes, including *SMAD2*, *ASCL1*, *NFIA*, and *E2F7* [65].

It has been reported that SRC-1 is significantly associated with disease recurrence in HER2-positive breast cancer patients [66]. Ets1 and Ets2, which are mitogen-activated protein kinase-activated transcription factors, bind to DNA response elements and recruit SRC-1 to recruit the transcription factor-DNA complex to upregulate HER2 protein expression [67]. The growth factor EGF induces transcription of Ets2-initiating oncogenes in a SRC-1-dependent manner; in this process, SRC-1 directly interacts with Ets2 to upregulate the expression of *c-Myc* and *MMP9* [68, 69]. MMTV-polyoma middle T antigen (PyMT) mice, which spontaneously develop breast tumours, were used to investigate the role of SRC-1 on tumour viability in vivo. One study showed that SRC-1 did not affect the proliferation of primary breast cancer cells but significantly promoted their invasion and lung metastasis [70]. SRC-1 may contribute to the metastatic activity of breast cancer by promoting Ets2-mediated HER2 expression and promoting the recruitment of macrophages to tumour sites by upregulating the expression of colony-stimulating factor 1 (CSF-1) [70]. The underlying mechanism was subsequently elucidated. SRC-1 and c-Fos can be recruited to a functional AP-1 site in the CSF-1 promoter, directly upregulating CSF-1 levels [71]. Angiogenesis is critical in the progression of breast cancer, and knockout of SRC-1 reduces the microvessel density (MVD) of breast cancer cells and inhibits angiogenesis in xenograft tumours, and these effects can be rescued by VEGFa treatment [72]. Mechanistically, SRC-1 promotes VEGFa transcription by associating with both c-Fos and HIF1α to recruit to the AP-1 site or HIF1α-binding element of the VEGFa promoter, respectively [72].

Furthermore, genetic deletion of SRC-1 can inhibit the migration and invasion of breast cancer cells by downregulating the expression of N-cadherin and vimentin and maintaining E-cadherin levels, and SRC-1 participates in the regulation of the above target proteins by cooperating with PEA3 to promote Twist expression [73]. Another possibility for metastatic breast cancer is that SRC-1 deficiency promotes the adhesion and migration of breast cancer cells to fibronectin, and further decreases the time needed for the degradation and reorganization of adhesions [74]. Integrin α5 (ITGA5) mediates cell adhesion and migration by upregulating paxillin, focal adhesion kinase, Rac1, and Erk1/2 expression or phosphorylation; SRC-1 can promote the transcriptional activity of the ITGA5 promoter by cooperating with the transcription factor AP-1 [74]. Leptin, derived from fat cells, stimulates the growth of breast epithelial cells and is a risk factor for breast cancer, especially in obese postmenopausal women. Leptin promotes breast cancer cell proliferation by activating STAT3; in this process, SRC-1 can be recruited to the STAT3 promoter and interact with its activation domain to enhance STAT3 signalling [75].

**Fig. 4:**
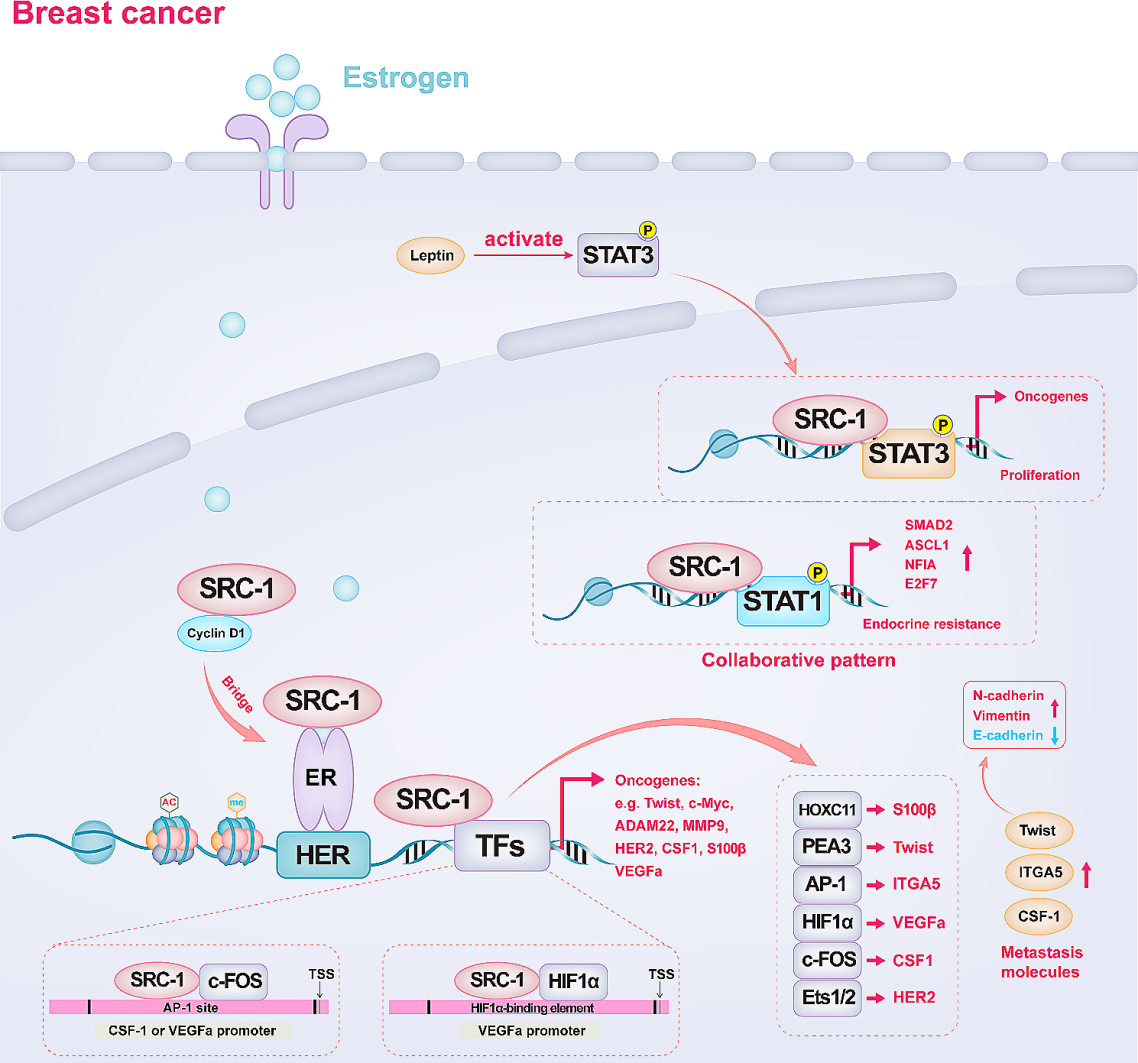
Potential mechanisms by which SRC-1 promotes breast cancer progression. The transactivation of ER is dependent on leucine-rich motifs, which constitute the ligand-regulated binding site of SRC-1. ER can interact with SRC-1 to modulate the expression of genes central to breast cancer progression. The transcriptional activity of several transcription factors, including HOXC11, PEA3, AP-1, HIF1α, c-FOS, Ets1/2, and STAT1/3, can be increased by SRC-1. Their target genes have various biological activities, such as promotion of tumour proliferation, metastasis, or angiogenesis

In conclusion, breast cancer is the primary cancer in which SRC-1 promotes progression. To better understand the mechanism by which SRC-1 promotes the progression of breast cancer, an overview map supported by existing evidence is presented (Fig. 4). Briefly, SRC-1 promotes the transcriptional activityof several transcription factors, including HOXC11, PEA3, AP-1, HIF1α, c-FOS, Ets1/2, and STAT1/3, in breast cancer. Their target genes have various forms of biological activity, such as promoting tumour proliferation, metastasis, or angiogenesis.

### Prostate cancer

**Fig. 5:**
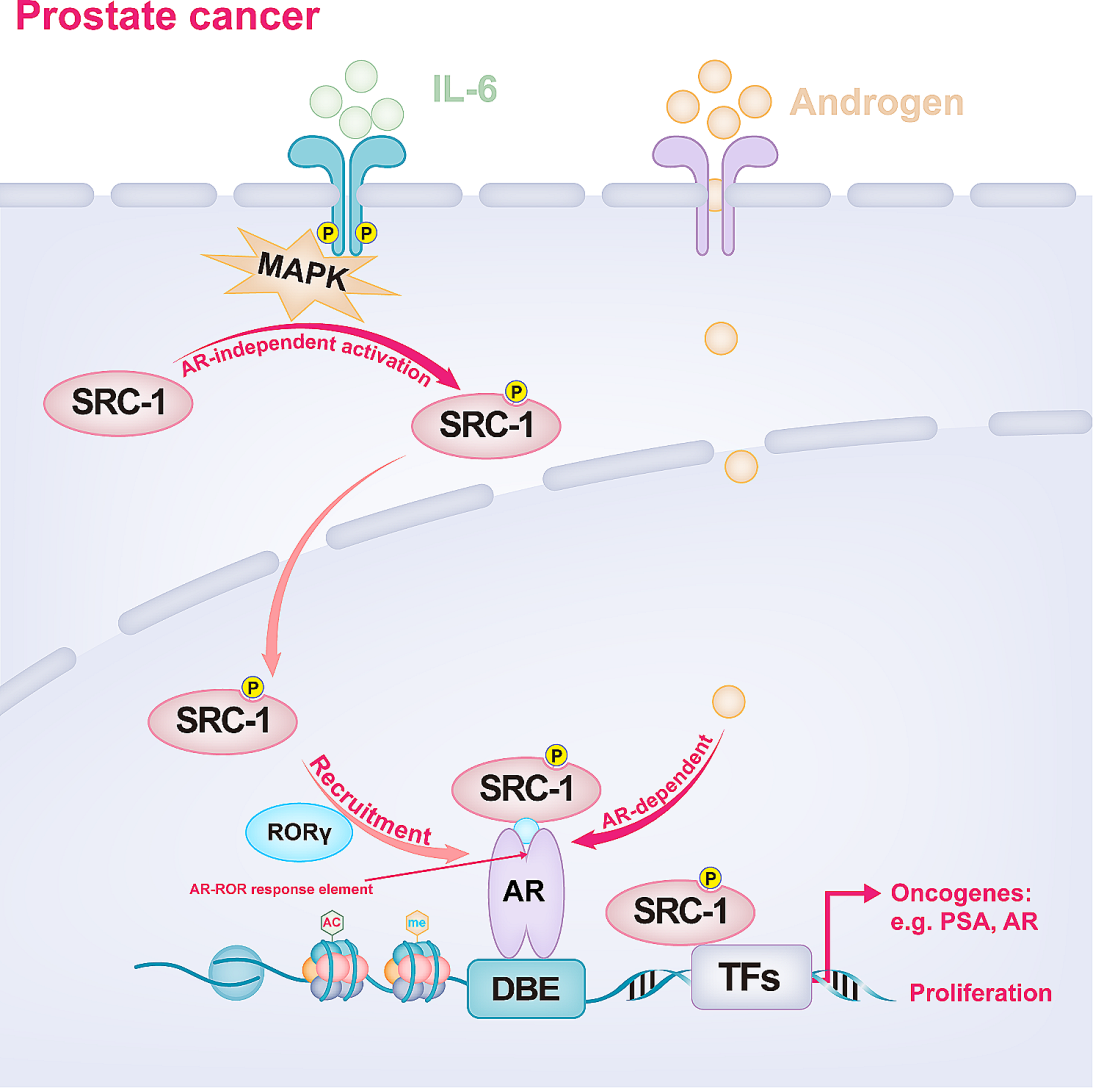
Potential mechanisms by which SRC-1 promotes prostate cancer progression. SRC-1 participates in androgen-induced AR activation and the regulation of several oncogenes related to prostate cancer progression. IL-6 can modulate AR-independent activation of SRC-1 and promote SRC-1 phosphorylation and nuclear transport by stimulating the MAPK pathway. RORγ can recruit SRC-1 to the AR-ROR response element to promote the transcriptional activity of AR-regulated genes

SRC-1 has the second-most potent effect in prostate cancer, as the prostate is rich in endogenous androgen, which regulates various physiological activities through the androgen receptor (AR), and SRC-1 is a crucial mediated of these effects. SRC-1 can functionally promote the transactivation of AR and participate in the ligand-independent activation of AR by IL-6 in prostate cancer cells [76, 77]. IL-6 mediates AR-independent activation in prostate cancer cells in the absence of androgens, and MAPK involvement is necessary. IL-6 promotes SRC-1 phosphorylation and nuclear transport by stimulating the MAPK pathway [77] (Fig. 5). Androgens in the epithelium and stroma drive the functional differentiation of the prostate epithelium as the critical process in the growth and development of prostate epithelial cells [78]. Ligands induce the binding of AR and SRC-1 to DNA elements in stromal cells, while epithelial cells promote this interaction between AR and SRC-1 in an androgen-dependent manner [78]. SRC-1 changes were found to be slight in normal prostate and prostate cancer tissues, but the increased expression of SRC-1 in tumour tissues was associated with the clinical and pathological variables of increased tumour invasiveness [79]. However, another study revealed that the expression of SRC-1 was significantly greater in primary prostate cancer tissue than in normal prostate tissue [80]. These distinct results may be related to tumour heterogeneity.

Furthermore, downregulation of SRC-1 expression inhibited the growth of prostate cancer cells and decreased the transcriptional activity of prostate-specific antigen (PSA), an AR target gene [79] (Fig. 5). However, some researchers believe that SRC-1 is not required for murine prostate carcinogenesis. They found that the expression of SRC-1 was relatively constant in mice with spontaneous prostate cancer, while that of SRC-3 was significantly increased; moreover, SRC-3 is overexpressed during prostate tumour progression in SRC-1-deficient mice, so SRC-3 may compensate for the loss of SRC-1 function [81]. Retinoic acid receptor-related orphan receptor γ (RORγ) is overexpressed and drives AR expression in castration-resistant prostate cancer (CRPC). RORγ can recruit SRC-1 to the AR-ROR response element to promote the transcriptional activation of AR-regulated genes [82] (Fig. 5).

### Gastrointestinal cancer

**Fig. 6:**
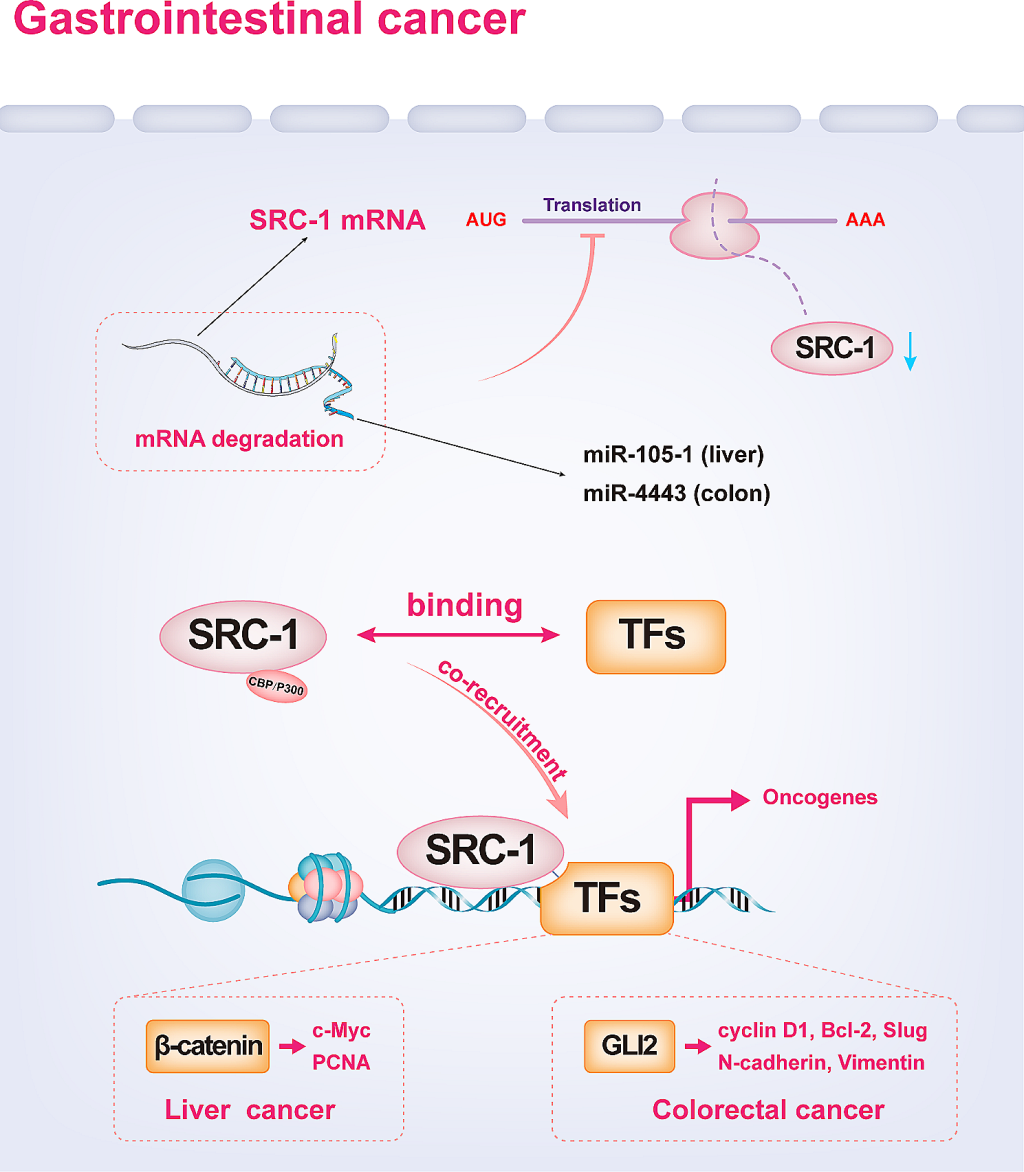
The potential role of SRC-1 in promoting the progression of liver and colorectal cancer. SRC-1 promotes the expression of c-Myc and PCNA by enhancing Wnt/β-catenin signalling, and miR-105-1 negatively regulates SRC-1 by binding to the 3’-UTR of SRC-1 mRNA in HCC. Similarly, SRC-1 promotes CRC progression by promoting the transcription of GLI2 target genes, and miR-4443 inhibits CRC cell proliferation and invasion through the negative regulation of SRC-1

The tumorigenic role of SRC-1 in human hepatocellular carcinoma (HCC) and colorectal cancer (CRC) has been demonstrated [16, 83]. SRC-1 was reported to be highly expressed in HCC tissue, with a positive rate of 62.5%, and knocking down SRC-1 can inhibit the proliferation of liver cancer cells and the growth of xenograft tumours in mice [83]. Activated Wnt/β-catenin signalling is critical and can be enhanced by SRC-1 via direct interaction with β-catenin, thereby promoting the expression of c-Myc and PCNA [83] (Fig. 6). Leupaxin, a novel coactivator of β-catenin involved in the promotion of HCC progression, can interact with β-catenin and promote its transcriptional activity by recruiting SRC-1 and p300 [84]. It has been reported that the microRNA miR-105-1 can negatively regulate SRC-1 to suppress the progression of HCC by binding to the 3’-UTR of SRC-1 mRNA to inhibit its expression [85] (Fig. 6). Another study indicated that the microRNA miR-4443 can be significantly upregulated by leptin and insulin in HCT116 and HT29 cells and can directly negatively regulate SRC-1 to inhibit the invasion and proliferation of CRC cells [86] (Fig. 6). Recently, the facilitative role of SRC-1 in CRC proliferation and metastasis has been comprehensively elucidated. SRC-1 is highly expressed in the tumour tissues of CRC patients; knockdown of SRC-1 significantly inhibits the proliferation and invasion of CRC cells in vitro, as well as their growth and metastasis in vivo [16]. GLI family zinc finger 2 (GLI2) is an important transcription factor in the Hedgehog signalling pathway that mediates the transcription of genes related to proliferation and invasion, including *cyclin D1*, *Bcl-2*, *Slug*, and *VIM*; SRC-1 can directly bind to the zinc finger domain of GLI2, co-recruit to their target gene promoter and serve as a coactivator to enhance the transcriptional activity of GLI2 [16] (Fig. 6). SRC-1 also has tumour-promoting effects in esophageal squamous cell carcinoma (ESCC). SRC-1 can affect the prognosis of ESCC and serve as an independent predictor of overall survival; knocking down SRC-1 can significantly inhibit the proliferation, migration, and invasion of ESCC cells [87].

### Neurological cancer

Meningiomas are more common in women than in men and are associated with progesterone receptor expression and hormone disruption. SRC-1 is expressed in meningioma patient tissues, and significantly associated with progesterone; progesterone is involved in the regulation of specific intracellular receptor interactions and SRC-1 is required for its transcriptional activation [88]. SRC-1 in astrocytoma is regulated by sex steroid hormones, and progesterone dynamically regulates SRC-1 expression at the evolutionary level in astrocytes [89]. Multiple factors are involved in the progression of astrocytoma; high expression of EGFR, cyclin D1, VEGF, and PR are features of this disease [90]. Progesterone treatment increased VEGF and EGFR levels in astrocytoma by acting on PR, and knockdown of SRC-1 significantly reduced VEGF expression but had no significant effect on EGFR [91]. Estradiol induces astrocytoma cell growth by acting on ERα, SRC-1 facilitates these effects [92]. Glioma is another major primary brain tumour that is associated with abnormal angiogenesis and abnormal proliferation of glioblastoma. SRC-1 can enhance angiogenesis mediated by basic fibroblast growth factor (bFGF) in vivo to promote glioma progression; in addition, SRC-1 binds with polyoncovirusenhancer activator 3 (PEA3) to promote the transcription of its target genes *VEGF* and *MMP-9*, regulating endothelial cell (EC) function and promoting angiogenesis [93]. Another study confirmed that SRC-1 could promote the proliferation, migration, and tumour growth of glioblastoma and was positively correlated with the grade of glioma but negatively correlated with the prognosis of glioma [94]. The team demonstrated that SRC-1 could promote the stemness of glioblastoma cells, possibly through the X-inactive specific transcript (XIST)/mi-152 axis, promoting the expression of Kruppel-like factor 4 (KLF4) [94]. Furthermore, some case reports suggest that SRC-1 positivity may be associated with the occurrence of ependymoma, but the underlying mechanism remains unclear [95, 96].

### Genital system cancer in females

In addition to breast cancer, tumours of the female genital system, including the uterus and ovaries, are also major causes of death, and the role of SRC-1 in these cancers has been reported. SRC-1 is highly expressed in normal endometrial tissues, with an average positive rate of 81.8%; unexpectedly, its expression is reduced in endometrial hyperplasia (58.9%) and endometrial carcinoma (45.0%) tissues [97]. A lack of mitogen-inducible gene 6 (Mig-6) has been reported to be associated with endometrial hyperplasia in mice and the progression of endometrial carcinoma; notably, this gene is regulated by SRC-1 [98]. The above studies indicate that SRC-1 may play a protective role in endometrial cancer but not in ovarian cancer. Growing evidence suggests that estrogen, which can induce the expression of c-Myc and IGF-1 and facilitate the binding of ERα to the AP1 site of the IGF-1 and c-Myc promoters to promote ovarian cancer cell proliferation, is a risk factor for epithelial ovarian cancer, and silencing SRC-1 can block inducible c-Myc expression and cell cycle progression [99].

### Other cancers

SRC-1 is less commonly associated with other common cancers, including melanoma, lung cancer, thyroid cancer, and lymphatic cancer, and reports are sporadic. SRC-1 and its partner HOXC11 are highly expressed in malignant melanoma, and SRC-1 can cooperate with HOXC11 to promote the expression of S100beta, which is a stimulating factor for cell proliferation and migration and an inhibitor of cell apoptosis and differentiation [100]. Estrogen and SRC-1 are intricately intertwined, and some researchers have explored their role in the progression of thyroid cancer, although the thyroid is not a direct target of estrogen. Estrogen promotes SRC-1 and cyclin D1 expression and the proliferation of thyroid cancer cells, but knockout of SRC-1 did not abolish estrogen-induced cell proliferation; a study also revealed that 87% of anaplastic thyroid cancer patients were SRC-1-positive, and SRC-1 positivity was associated with reduced disease-free survival [101]. Another study reported that SRC-1 protein levels were elevated in thyroid cancer tissues compared to normal thyroid tissues. The team found that the expression of SRC-1 is positively correlated with vascular endothelial growth factor C (VEGFC) and that SRC-1 can bind with NF-κB to form a coactivating complex that directly promotes VEGFC transcription, and this effect can be abolished by knocking down SRC-1 [102]. Although the incidence rate of lung cancer is high, the tumorigenic role of SRC-1 in lung cancer remains unclear.

## SRC-1-targeted therapy

To date, a variety of small molecule inhibitors, including peptides, natural compounds, artificial small molecules, and naturally active extracts, have been reported to decrease the activity of SRC-1 directly or indirectly. A peptide containing the LXXLL motif of human SRC-1 (TP10-SRC1LXXLL) induced the dose-dependent death of breast cancer cells in an ER- and hormone-independent manner [103]. The flavonoid 3,6-dihydroxyflavone, which promotes the binding between PPARγ and SRC-1, can activate hPPAR and has a cytotoxic effect on human cervical cancer and prostate cancer cells [104]. Another study indicated that two naturally occurring sesquiterpenoids (ST1 and ST2) could inhibit the expression of SRC-1 and AR in prostate cancer cells and suppress the nuclear transport of AR, further inhibiting the interaction between SRC-1 and AR [105]. Metformin, a well-known antidiabetic drug, has been reported to inhibit the androgen-dependent proliferation of prostate cancer cells by inhibiting the function of AR and the expression of its target genes; in this process, small heterodimer partner-interacting leucine zipper (SMILE), which is a nuclear receptor coregulator whose protein level can be increased by metformin, competes with SRC-1 to bind AR to inhibit SRC-1-mediated transactivation [106].

Bufalin is a cardiotonic agent extracted from the skin secretions of toads and has anticancer activity. It has been reported that bufalin can induce caspase-mediated cell apoptosis and decrease the levels of SRC-1, AR, and its target gene PSA, exerting an anti-prostate cancer effect [107]. Another study indicated that bufalin could inhibit CRC progression by inhibiting SRC-1 and its regulated Hedgehog signalling; in particular, the authors demonstrated that bufalin could increase the therapeutic efficacy of the Hedgehog pathway-targeting drug vismodegib in CRC [16]. Moreover, bufalin can reduce glioblastoma viability by inhibiting SRC-1 [94]. Additionally, dasatinib, an ATP-competitive dual Src/Abl inhibitor, could reverse SRC-1-mediated melanoma progression by suppressing the interaction between HOXC11 and SRC-1 [100].

## Conclusions

The emerging multiorgan oncogenic role of SRC-1 is still being studied, especially its roles in promoting breast cancer, prostate cancer, gastrointestinal cancer, neurological cancer, and genital system cancer. The breast and prostate are the main affected organs because of their rich steroid hormone production. SRC-1 serves as a coactivator of multiple transcription factors, such as HOXC11, PEA2, AP-1, HIF1α, c-FOS, Ets1/2, and STAT1/3, to promote the expression of oncogenes, including *S100β*, *Twist*, *ITGA5*, *VEGFa*, *CSF1*, and *HER2*, in the progression of breast cancer, and estrogen-mediated ER activation is critical (Fig. 4). Similarly, androgen-mediated AR activation and SRC-1 recruitment are also classic mechanisms involved in prostate carcinogenesis (Fig. 5). In addition, clear evidence indicates that SRC-1 promotes liver and colorectal cancer progression by synergistically promoting Wnt/β-catenin or Hedgehog pathway signalling, respectively, possibly through a steroid hormone-independent pathway (Fig. 6). The tumorigenic role of SRC-1 varies in organs such as the uterus, ovaries, brain, skin, and thyroid, so further study is needed. Although a variety of targeted inhibitors of SRC-1 are available, their practical application in clinical cancer therapy is very rare. Therefore, research on the tumorigenic roles of SRC-1 needs further improvement, and we sincerely hope that our review will attract more researchers to investigate this topic.

## Data Availability

Not applicable.

## Abbreviations

bHLH-PAS: basic helix-loop-helix/Per/ARNT/Sim domain
NRID: nuclear receptor interaction domain
CBP: CREB-binding protein
CID: CBP interacting domain
HAT: histone acetyltransferase
AD1/2: activation domain 1/2
NRs: nuclear receptors
PRMT1: protein arginine N-methyltransferase 1
CARM1: coactivator-associated arginine methyltransferase 1
PPARγ: peroxisome proliferator-activated receptorγ
ER: estrogen receptor
PR: progestins receptor
SP-A: Surfactant protein-A
Il13ra2: Interleukin-13 receptor subunit alpha-2
PGF2α: prostaglandin F2 alpha
Pomc: pro-opiomelanocortin
HOXC11: Homeobox protein Hox-C11
PEA3: polyoncovirus enhancer activator 3
Twist: Twist-related protein
ITGA5: integrin alpha-5
CSF-1: colony-stimulating factor 1
SMAD2: mothers against decapentaplegic homolog 2
ASCL1: Achaete-scute homolog 1
NFIA: Nuclear factor 1 A-type
E2F7: E2F transcription factor 7
c-FOS: cellular oncogene fos (AP-1 transcription factor subunit)
HIF1α: hypoxia-inducible factor 1-alpha
MAPK: mitogen-activated protein kinase
RORγ: retinoic acid receptor-related orphan receptorγ
AR: androgen receptor
PSA: prostate-specific antigen
DBE: DNA binding element
GLI2: GLI family zinc finger protein 2

## References

[1] Meng Z, Wang X;ZhangD, ;Lan Z, Cai X, Bian C, Zhang J (2022). Steroid receptor coactivator-1: the central intermediator linking multiple signals and functions in the brain and spinal cord. Genes Dis.

[2] Onate SA, Spencer TE, Edwards DP, O’Malley BW (1998). The steroid receptor coactivator-1 contains multiple receptor interacting and activation domains that cooperatively enhance the activation function 1 (AF1) and AF2 domains of steroid receptors. J Biol Chem.

[3] McInerney EM, Rose DW, Flynn SE, Mullen TM, Inostroza J, Torchia J, Nolte RT, Assa-Munt N (1998). Determinants of coactivator LXXLL motif specificity in nuclear receptor transcriptional activation. Genes Dev.

[4] Rohira AD (2017). Steroid receptor coactivators present a unique opportunity for drug development in hormone-dependent cancers. Biochem Pharmacol.

[5] Xu J, Wu RC, O’Malley BW (2009). Normal and cancer-related functions of the p160 steroid receptor co-activator (SRC) family. Nat Rev Cancer.

[6] Spencer TE, Burcin MM, Allis CD, Mizzen CA, Onate SA, Tsai SY, Tsai MJ (1997). Steroid receptor coactivator-1 is a histone acetyltransferase. Nature.

[7] Chen H, Lin RJ, Nash A, Nagy L, Privalsky ML, Evans RM (1997). Nuclear receptor coactivator ACTR is a novel histone acetyltransferase and forms a multimeric activation complex with P/CAF and CBP/p300. Cell.

[8] Oñate SA, O’Malley BW (1995). Sequence and characterization of a coactivator for the steroid hormone receptor superfamily. Science.

[9] Voegel JJ, Heine MJ, Chambon P, ;Gronemeyer H (1996). TIF2, a 160 kDa transcriptional mediator for the ligand-dependent activation function AF-2 of nuclear receptors. Embo j.

[10] Torchia J, Rose DW, Kamei Y, Westin S, Glass CK (1997). .;Rosenfeld M.G. The transcriptional co-activator p/CIP binds CBP and mediates nuclear-receptor function. Nature.

[11] Li H, Gomes PJ (1997). RAC3, a steroid/nuclear receptor-associated coactivator that is related to SRC-1 and TIF2. Proc Natl Acad Sci U S A.

[12] Anzick SL, Walker RL, Kallioniemi OP, Trent JM (1997). Meltzer P.S. AIB1, a steroid receptor coactivator amplified in breast and ovarian cancer. Science.

[13] Takeshita A, Cardona GR, Suen CS, Chin WW (1997). TRAM-1, a novel 160-kDa thyroid hormone receptor activator molecule, exhibits distinct properties from steroid receptor coactivator-1. J Biol Chem.

[14] Gilad Y, Lonard DM, O’Malley BW (2022). Steroid receptor coactivators - their role in immunity. Front Immunol.

[15] McKenna NJ, Tsai SY, O’Malley BW (1998). Distinct steady-state nuclear receptor coregulator complexes exist in vivo. Proc Natl Acad Sci U S A.

[16] Guo P, Chen Q, ;Peng K, Xie J, Liu J, Ren W, Tong Z, Li M, Xu J, Zhang Y (2022). Nuclear receptor coactivator SRC-1 promotes colorectal cancer progression through enhancing GLI2-mediated hedgehog signaling. Oncogene.

[17] Jain S, Pulikuri S, Zhu Y, Qi C, Kanwar YS (1998). Differential expression of the peroxisome proliferator-activated receptor gamma (PPARgamma) and its coactivators steroid receptor coactivator-1 and PPAR-binding protein PBP in the brown fat, urinary bladder, colon, and breast of the mouse. Am J Pathol.

[18] Zhu Y, Qi C, Jain S, Rao MS (1997). Isolation and characterization of PBP, a protein that interacts with peroxisome proliferator-activated receptor. J Biol Chem.

[19] Chen S, Johnson BA, Aster S, Mosley R, Moller DE (2000). Both coactivator LXXLL motif-dependent and -independent interactions are required for peroxisome proliferator-activated receptor gamma (PPARgamma) function. J Biol Chem.

[20] Cho MC, Lee WS, Hong JT, Park SW, Paik SG (2005). 5-(3,5-Di-tert-butyl-4-hydroxybenzylidene) thiazolidine-2,4-dione modulates peroxisome proliferators-activated receptor gamma in 3T3-L1 adipocytes: roles as a PPARgamma ligand. Mol Cell Endocrinol.

[21] Qi C, Zhu Y, ;Pan J, Yeldandi AV (1999). Maeda N.;Subbarao V.;Pulikuri S.;Hashimoto T.;Reddy J.K. Mouse steroid receptor coactivator-1 is not essential for peroxisome proliferator-activated receptor alpha-regulated gene expression. Proc Natl Acad Sci U S A.

[22] Mottillo EP, Yang A, Zhou L, Granneman JG (2019). Genetically-encoded sensors to detect fatty acid production and trafficking. Mol Metab.

[23] Picard F, Annicotte J, Rocchi S, Champy MF, O’Malley BW (2002). Chambon P.;Auwerx J. SRC-1 and TIF2 control energy balance between white and brown adipose tissues. Cell.

[24] Shen L, Liu Y, ;Tso P, Wang DQ, Woods SC (2018). Silencing steroid receptor coactivator-1 in the nucleus of the solitary tract reduces estrogenic effects on feeding and apolipoprotein A-IV expression. J Biol Chem.

[25] Yamamuro T, Nakamura S, Yanagawa K, Tokumura A (2022). Kawabata T.;Fukuhara A.;Teranishi H.;Hamasaki M.;Shimomura I.;Yoshimori T. Loss of RUBCN/rubicon in adipocytes mediates the upregulation of autophagy to promote the fasting response. Autophagy.

[26] Zhou B, Jia L, ;Zhang Z, Xiang L, Zheng P, Liu B et al. Ren X.;Bian H.;Xie L.,. The Nuclear Orphan Receptor NR2F6 Promotes Hepatic Steatosis through Upregulation of Fatty Acid Transporter CD36. *Adv Sci (Weinh)*. 2020, 7, 2002273. 10.1002/advs.202002273. 10.1002/advs.202002273 PMC7610302 33173745

[27] Costantino S, Paneni F, Virdis A, Hussain S, Mohammed SA, Akhmedov A, Dalgaard K, Pospisilik JA (2019). Interplay among H3K9-editing enzymes SUV39H1, JMJD2C and SRC-1 drives p66Shc transcription and vascular oxidative stress in obesity. Eur Heart J.

[28] Tannour-Louet M, York B, Tang K, Stashi E, Bouguerra H, Zhou S, Yu H, Wong LJ, Stevens RD (2014). Hepatic SRC-1 activity orchestrates transcriptional circuitries of amino acid pathways with potential relevance for human metabolic pathogenesis. Mol Endocrinol.

[29] Louet JF, Chopra AR, York B, Tannour-Louet M, Saha PK, Wenner BR, Ilkayeva OR (2010). The coactivator SRC-1 is an essential coordinator of hepatic glucose production. Cell Metab.

[30] Motamed M, Rajapakshe KI, Moses RE, O’Malley BW (2014). Steroid receptor coactivator 1 is an integrator of glucose and NAD+/NADH homeostasis. Mol Endocrinol.

[31] Ahima RS;Mantzoros C.;Qu D.;Lowell, Maratos-Flier B, Flier E. J.S. Role of leptin in the neuroendocrine response to fasting. *Nature*. 1996, 382, 250–252. 10.1038/382250a0. 10.1038/382250a0 8717038

[32] van der Yang Y. Zhu L;Cacciottolo TM;He Y;Stadler LKJ;Wang C;Xu P;Saito K;Hinton A Jr, Steroid receptor coactivator-1 modulates the function of Pomc neurons and energy homeostasis. *Nat Commun*. 2019, 10, 1718. 10.1038/s41467-019-08737-6. 10.1038/s41467-019-08737-6 PMC6461669 30979869

[33] Bian C, Huang Y, ;Zhu H, Zhao Y, Zhao J;ZhangJ (2018). Steroid receptor Coactivator-1 knockdown decreases synaptic plasticity and impairs spatial memory in the Hippocampus of mice. Neuroscience.

[34] Chen X, Tian Y, Zhu H, Bian C, Li M (2020). Inhibition of steroid receptor coactivator-1 in the hippocampus impairs the consolidation and reconsolidation of contextual fear memory in mice. Life Sci.

[35] Molenda-Figueira HA, Murphy SD, Chadwick JG (2008). Jr.;Denner L.A.;Tetel M.J. Steroid receptor coactivator-1 from brain physically interacts differentially with steroid receptor subtypes. Endocrinology.

[36] Tognoni CM (2011). Nuclear receptor coactivators are coexpressed with steroid receptors and regulated by estradiol in mouse brain. Neuroendocrinology.

[37] Xiao J, ;Zhang J, Zhao Y, Huang W, ;Guo Z, Su B, Guo Q (2017). Sex differences of steroid receptor coactivator-1 expression after spinal cord injury in mice. Neurol Res.

[38] Bian C, Zhang D, Guo Q, Cai W, Zhang J (2011). Localization and sex-difference of steroid receptor coactivator-1 immunoreactivities in the brain of adult female and male mice. Steroids.

[39] Zhang D, Guo Q, Bian C, Zhang J, ;Lin S, Su B (2011). Alterations of steroid receptor coactivator-1 (SRC-1) immunoreactivities in specific brain regions of young and middle-aged female Sprague-Dawley rats. Brain Res.

[40] Bian C, Zhao Y, Guo Q, ;Xiong Y, Cai W (2014). Aromatase inhibitor letrozole downregulates steroid receptor coactivator-1 in specific brain regions that primarily related to memory, neuroendocrine and integration. J Steroid Biochem Mol Biol.

[41] Liu M, Huangfu X, Zhao Y, Zhang D (2015). Steroid receptor coactivator-1 mediates letrozole induced downregulation of postsynaptic protein PSD-95 in the hippocampus of adult female rats. J Steroid Biochem Mol Biol.

[42] Zhao Y, Zhao J, Liu Z, Xing F, Liu M, Feng Z, Li W (2017). Estrogen receptor alpha and beta regulate actin polymerization and spatial memory through an SRC-1/mTORC2-dependent pathway in the hippocampus of female mice. J Steroid Biochem Mol Biol.

[43] Xing FZ, Zhao JK, Zhang JQ (2018). Nuclear and membrane estrogen receptor antagonists induce similar mTORC2 activation-reversible changes in synaptic protein expression and actin polymerization in the mouse hippocampus. CNS Neurosci Ther.

[44] Zhang YY, Zhao JK, Zhang JQ (2019). GPR30-mediated estrogenic regulation of actin polymerization and spatial memory involves SRC-1 and PI3K-mTORC2 in the hippocampus of female mice. CNS Neurosci Ther.

[45] Zhao Y, Yu Y, He L, ;Qiu L, Zhao J, Liu M, Zhang J (2017). Letrozole regulates actin cytoskeleton polymerization dynamics in a SRC-1 dependent manner in the hippocampus of mice. J Steroid Biochem Mol Biol.

[46] Qiu L, Zhao Y, Guo Q, Zhang Y, He L, Zhang J (2016). Dose-dependent regulation of steroid receptor coactivator-1 and steroid receptors by testosterone propionate in the hippocampus of adult male mice. J Steroid Biochem Mol Biol.

[47] Zhao J, Bian C, Liu M, Zhao Y, Sun T, Zhang J (2018). Orchiectomy and letrozole differentially regulate synaptic plasticity and spatial memory in a manner that is mediated by SRC-1 in the hippocampus of male mice. J Steroid Biochem Mol Biol.

[48] Condon JC, Faust JM (2004). Surfactant protein secreted by the maturing mouse fetal lung acts as a hormone that signals the initiation of parturition. Proc Natl Acad Sci U S A.

[49] Gao L, Rabbitt EH, Condon JC, Renthal NE, Mitsche MA, Xu J, O’Malley BW (2015). Mendelson C.R. Steroid receptor coactivators 1 and 2 mediate fetal-to-maternal signaling that initiates parturition. J Clin Invest.

[50] Ou CW, Sadej W, Gibb W. Expression of Nuclear Receptor Coactivators in the Human Fetal Membranes at Term before and after Labor. *Obstet Gynecol Int*. 2012, 2012, 717294. 10.1155/2012/717294. 10.1155/2012/717294 PMC3539340 23316238

[51] Marquardt RM, Kim TH (2020). Interleukin-13 receptor subunit alpha-2 is a target of progesterone receptor and steroid receptor coactivator-1 in the mouse uterus†. Biol Reprod.

[52] Peter I, Shearman AM, Schmid CH, Cupples LA, D’Agostino RB, Karas RH (2005). Variation in estrogen-related genes and cross-sectional and longitudinal blood pressure in the Framingham Heart Study. J Hypertens.

[53] Hinton AO, Jr.;Yang Y, Quick AP, Reddy CL, Reynolds CL, Zhu L, Xu J (2016). SRC-1 regulates blood pressure and aortic stiffness in female mice. PLoS ONE.

[54] Paneni F, Beckman JA, Creager MA (2013). Diabetes and vascular disease: pathophysiology, clinical consequences, and medical therapy: part I. Eur Heart J.

[55] Yuan Y, Xu J (2007). Loss-of-function deletion of the steroid receptor coactivator-1 gene in mice reduces estrogen effect on the vascular injury response. Arterioscler Thromb Vasc Biol.

[56] Berns EM, Klijn JG (1998). Predictive value of SRC-1 for tamoxifen response of recurrent breast cancer. Breast Cancer Res Treat.

[57] Redmond AM, Bane FT, Stafford AT, Dillon MF, Crotty TB (2009). .;Young L.S. Coassociation of estrogen receptor and p160 proteins predicts resistance to endocrine treatment; SRC-1 is an independent predictor of breast cancer recurrence. Clin Cancer Res.

[58] Zhang H, Yi X (2004). Sun X.;Yin N.;Shi B.;Wu H.;Wang D.;Wu G.;Shang Y. Differential gene regulation by the SRC family of coactivators. Genes Dev.

[59] Myers E, Fleming FJ, Crotty TB, McDermott EW, O’Higgins NJ, Hill AD (2004). Young L.S. Inverse relationship between ER-beta and SRC-1 predicts outcome in endocrine-resistant breast cancer. Br J Cancer.

[60] Zwijsen RM, Buckle RS, Loomans CJ (1998). Ligand-independent recruitment of steroid receptor coactivators to estrogen receptor by cyclin D1. Genes Dev.

[61] Kishimoto H, Wang Z, Bhat-Nakshatri P, Chang D, Clarke R, Nakshatri H (2005). The p160 family coactivators regulate breast cancer cell proliferation and invasion through autocrine/paracrine activity of SDF-1alpha/CXCL12. Carcinogenesis.

[62] McCartan D, Bolger JC, Byrne C, ;Hao Y, Qin L, Xu J, Hill AD (2012). Global characterization of the SRC-1 transcriptome identifies ADAM22 as an ER-independent mediator of endocrine-resistant breast cancer. Cancer Res.

[63] McIlroy M, P O.G.;Pennington S, Hill AD (2010). Interaction of developmental transcription factor HOXC11 with steroid receptor coactivator SRC-1 mediates resistance to endocrine therapy in breast cancer [corrected]. Cancer Res.

[64] Ward E, ;Charmsaz S, Fagan A, Browne AL, Cocchiglia S, Purcell SP, Das S (2018). Epigenome-wide SRC-1-Mediated gene silencing represses Cellular differentiation in advanced breast Cancer. Clin Cancer Res.

[65] Browne AL, Charmsaz S, Varešlija D, Fagan A, Cosgrove N, Cocchiglia S, Purcell S, Ward E, Bane F, Hudson L (2018). Network analysis of SRC-1 reveals a novel transcription factor hub which regulates endocrine resistant breast cancer. Oncogene.

[66] Fleming FJ, Kelly G, Crotty TB, O’Higgins NJ, Hill AD (2004). Expression of SRC-1, AIB1, and PEA3 in HER2 mediated endocrine resistant breast cancer; a predictive role for SRC-1. J Clin Pathol.

[67] Myers E, Hill AD, McDermott EW, O’Higgins NJ, Buggy Y, Young LS (2005). Associations and interactions between Ets-1 and Ets-2 and coregulatory proteins, SRC-1, AIB1, and NCoR in breast cancer. Clin Cancer Res.

[68] Al-azawi D, Redmond AM, Bane FT, Hill AD (2008). Ets-2 and p160 proteins collaborate to regulate c-Myc in endocrine resistant breast cancer. Oncogene.

[69] McBryan J, Theissen SM, Hughes E, Cocchiglia S, Sande S, O’Hara J, Tibbitts P, Hill AD (2012). Young L.S. Metastatic progression with resistance to aromatase inhibitors is driven by the steroid receptor coactivator SRC-1. Cancer Res.

[70] Wang S, Yuan Y, Liao L, Kuang SQ, O’Malley BW (2009). Disruption of the SRC-1 gene in mice suppresses breast cancer metastasis without affecting primary tumor formation. Proc Natl Acad Sci U S A.

[71] Qin L, Wu YL, Liao L, Gao X, Bane FT, Xu Y, Feng Z (2014). NCOA1 directly targets M-CSF1 expression to promote breast Cancer metastasis. Cancer Res.

[72] Qin L, Xu Y, Ma G, ;Liao L, Li Y, Wang X, Jiang J (2015). NCOA1 promotes angiogenesis in breast tumors by simultaneously enhancing both HIF1α- and AP-1-mediated VEGFa transcription. Oncotarget.

[73] Qin L, ;Liu Z, Chen H (2009). The steroid receptor coactivator-1 regulates twist expression and promotes breast cancer metastasis. Cancer Res.

[74] Qin L, ;Chen X, Wu Y, Feng Z, He T, Wang L, Xu J (2011). Steroid receptor coactivator-1 upregulates integrin α_5_ expression to promote breast cancer cell adhesion and migration. Cancer Res.

[75] Yin N, Wang D, Zhang H, Yi X (2004). Sun X.;Shi B.;Wu H.;Wu G.;Wang X.;Shang Y. Molecular mechanisms involved in the growth stimulation of breast cancer cells by leptin. Cancer Res.

[76] Yeh S, Miyamoto H, Shima H, Chang C (1998). From estrogen to androgen receptor: a new pathway for sex hormones in prostate. Proc Natl Acad Sci U S A.

[77] Ueda T, Mawji NR, Bruchovsky N, Sadar MD (2002). Ligand-independent activation of the androgen receptor by interleukin-6 and the role of steroid receptor coactivator-1 in prostate cancer cells. J Biol Chem.

[78] Cano P, Godoy A, Escamilla R, Dhir R, Onate SA (2007). Stromal-epithelial cell interactions and androgen receptor-coregulator recruitment is altered in the tissue microenvironment of prostate cancer. Cancer Res.

[79] Agoulnik IU, Bingman WE 3rd, ;Erdeme H, Frolov A, Smith CL, Ittmann MM. Weigel N.L. Role of SRC-1 in the promotion of prostate cancer cell growth and tumor progression. Cancer Res. 2005;65:7959–67. 10.1158/0008-5472.can-04-3541. 10.1158/0008-5472.CAN-04-3541 16140968

[80] Luef B, Handle F, Kharaishvili G;Hager M.;Rainer J.;Janetschek G.;Hruby S.;Englberger C.;Bouchal J.;, Santer FR et al. The AR/NCOA1 axis regulates prostate cancer migration by involvement of PRKD1. *Endocr Relat Cancer*. 2016, 23, 495–508. 10.1530/erc-16-0160. 10.1530/ERC-16-0160 27255895

[81] Tien JC, Xu J (2009). The role of SRC-1 in murine prostate cancinogenesis is nonessential due to a possible compensation of SRC-3/AIB1 overexpression. Int J Biol Sci.

[82] Wang J, Zou JX, Cai D, Zhang Y, Duan Z, Xiang Q, Yang JC, Borowsky AD (2016). ROR-γ drives androgen receptor expression and represents a therapeutic target in castration-resistant prostate cancer. Nat Med.

[83] Tong Z, Li M, Wang W, Mo P, Yu L, ;Liu K, Ren W, Li W, Zhang H, Xu J (2015). Steroid receptor coactivator 1 promotes human hepatocellular carcinoma progression by enhancing Wnt/β-Catenin signaling. J Biol Chem.

[84] Shi J, Wu WJ, Yu X, Yu GS, Yang ML (2016). Wu Z.X. Regulation of β-catenin transcription activity by leupaxin in hepatocellular carcinoma. Tumour Biol.

[85] Ma YS, Lv ZW (2017). High expression of mir-105-1 positively correlates with clinical prognosis of hepatocellular carcinoma by targeting oncogene NCOA1. Oncotarget.

[86] Meerson A, Yehuda H (2016). Leptin and insulin up-regulate miR-4443 to suppress NCOA1 and TRAF4, and decrease the invasiveness of human colon cancer cells. BMC Cancer.

[87] Wang L, Li W, Li K, Guo Y, Liu D, ;Yao Z, Lin X, Li S, Jiang Z (2018). The oncogenic roles of nuclear receptor coactivator 1 in human esophageal carcinoma. Cancer Med.

[88] Carroll RS, Zhang J, De Mora J (2000). Black P.M. expression of a subset of steroid receptor cofactors is associated with progesterone receptor expression in meningiomas. Clin Cancer Res.

[89] Hernández-Hernández OT, Rodríguez-Dorantes M, González-Arenas A, Camacho-Arroyo I (2010). Progesterone and estradiol effects on SRC-1 and SRC-3 expression in human astrocytoma cell lines. Endocrine.

[90] Louis DN (2006). Molecular pathology of malignant gliomas. Annu Rev Pathol.

[91] Hernández-Hernández OT, González-García TK, Camacho-Arroyo I (2012). Progesterone receptor and SRC-1 participate in the regulation of VEGF, EGFR and Cyclin D1 expression in human astrocytoma cell lines. J Steroid Biochem Mol Biol.

[92] González-Arenas A, Hansberg-Pastor V, Hernández-Hernández OT, González-García TK, Henderson-Villalpando J, Lemus-Hernández D, Cruz-Barrios A, Rivas-Suárez M, Camacho-Arroyo I (2012). Estradiol increases cell growth in human astrocytoma cell lines through ERα activation and its interaction with SRC-1 and SRC-3 coactivators. Biochim Biophys Acta.

[93] Zhang Y, Shi W (2019). Steroid receptor coactivator-1 regulates glioma angiogenesis through polyomavirus enhancer activator 3 signaling. Biochem Cell Biol.

[94] Gong M, Wang X;MuL, ;Wang Y, Pan J, Yuan X, Zhou H;XingJ, Wang R, Sun J et al. Steroid receptor coactivator-1 enhances the stemness of glioblastoma by activating long noncoding RNA XIST/miR-152/KLF4 pathway. 2021, 112, 604–18. 10.1111/cas.14685. 10.1111/cas.14685 PMC7894023 33090636

[95] Tomomasa R, Arai Y, Kawabata-Iwakawa R, Fukuoka K;Nakano Y.;Hama N.;Nakata S.;Suzuki N., ;Ishi Y, Tanaka S et al. Ependymoma-like tumor with mesenchymal differentiation harboring C11orf95-NCOA1/2 or -RELA fusion: A hitherto unclassified tumor related to ependymoma. 2021, 31, e12943. 10.1111/bpa.12943. 10.1111/bpa.12943 PMC8412126 33576087

[96] Tauziède-Espariat A, Siegfried A, ;Nicaise Y, Kergrohen T, ;Sievers P et al. Vasiljevic A.;Roux A.;Dezamis E.;Benevello C.;Machet M.C.,. Supratentorial non-RELA, ZFTA-fused ependymomas: a comprehensive phenotype genotype correlation highlighting the number of zinc fingers in ZFTA-NCOA1/2 fusions. *Acta Neuropathol Commun*. 2021, 9, 135. 10.1186/s40478-021-01238-y. 10.1186/s40478-021-01238-y PMC8362233 34389065

[97] Uchikawa J, Shiozawa T, Shih HC, Feng YZ (2003). Oka K.;Konishi I. expression of steroid receptor coactivators and corepressors in human endometrial hyperplasia and carcinoma with relevance to steroid receptors and Ki-67 expression. Cancer.

[98] Jeong JW, Lee KY, Vande Woude GF, Young SL (2009). Mig-6 modulates uterine steroid hormone responsiveness and exhibits altered expression in endometrial disease. Proc Natl Acad Sci U S A.

[99] Sasaki H, Hayakawa J, Terai Y, Kanemura M, Tanabe-Kimura A, Kamegai H, Seino-Noda H, Ezoe S, Matsumura I, Kanakura Y (2008). Difference between genomic actions of estrogen versus raloxifene in human ovarian cancer cell lines. Oncogene.

[100] deBlacam C, Byrne C, Hughes E, McIlroy M, Bane F, Hill AD, Young LS (2011). HOXC11-SRC-1 regulation of S100beta in cutaneous melanoma: new targets for the kinase inhibitor dasatinib. Br J Cancer.

[101] Kavanagh DO, Bane F, Crotty TB, Hill AD (2010). Young L.S. The role of oestrogen receptor {alpha} in human thyroid cancer: contributions from coregulatory proteins and the tyrosine kinase receptor HER2. Endocr Relat Cancer.

[102] Gao B, Guo L, ;Luo D, ;Jiang Y, Zhao J, Mao C, Xu Y. Steroid receptor coactivator-1 interacts with NF-κB to increase VEGFC levels in human thyroid cancer. Biosci Rep. 2018;38. 10.1042/bsr20180394. 10.1042/BSR20180394 PMC5997793 29717026

[103] Tints K, Prink M, Neuman T, Palm K (2014). LXXLL peptide converts transportan 10 to a potent inducer of apoptosis in breast cancer cells. Int J Mol Sci.

[104] Lee JY, Yoon DY (2010). Cytotoxic flavonoids as agonists of peroxisome proliferator-activated receptor gamma on human cervical and prostate cancer cells. J Nat Prod.

[105] Wang XL, Shen T, Young CY (2011). Sesquiterpenoids from myrrh inhibit androgen receptor expression and function in human prostate cancer cells. Acta Pharmacol Sin.

[106] Lee SY, Xie YB, Choi HS (2014). SMILE upregulated by metformin inhibits the function of androgen receptor in prostate cancer cells. Cancer Lett.

[107] Zhang Y, Dong Y, Melkus MW, Tang SN, Pramanik K, Wu W, Kim S (2018). Role of P53-Senescence induction in suppression of LNCaP prostate Cancer growth by Cardiotonic Compound Bufalin. Mol Cancer Ther.

